# A Multiplex TaqMan qPCR Assay for Rapid Detection of *Streptococcus suis* Serotypes 1, 2, 7, and 9 with Cross-Reactivity to Serotypes 14 and 1/2

**DOI:** 10.3390/vetsci13090884

**Published:** 2026-08-29

**Authors:** Jingye Huang, Tingting Yue, Jianing Huang, Qiong Chen, Min Zheng, Meiling Yu, Mingsheng Jiang, Xiulin Li, Hailan Chen

**Affiliations:** 1 Guangxi Key Laboratory of Animal Breeding, Disease Control and Prevention, College of Animal Science and Technology, Guangxi University, Nanning 530004, China; 2 Nanning Zhufulai Animal Health Management Co., Ltd., Nanning 530006, China; 3 Guangxi Center for Animal Disease Control and Prevention, Nanning 530001, China

**Keywords:** *Streptococcus suis*, zoonotic pathogen, multiplex qPCR, TaqMan assay, public health surveillance, serotype monitoring

## Abstract

*Streptococcus suis* (*S. suis*) is a zoonotic pathogen that causes severe diseases including meningitis, septic shock, and endocarditis in both pigs and humans. In swine, *S. suis* is one of the most important bacterial pathogens causing invasive diseases such as meningitis, septicemia, arthritis, and sudden death, with serotypes 1, 2, 7, and 9 being among the most frequently recovered from clinical cases in affected herds across Southeast Asia. We developed a multiplex TaqMan qPCR assay targeting four serotype-specific genes (*cps1I*, *cps2I*, *cps7L*, and *cps9J*) for detection of major *S. suis* serotypes in porcine samples. The assay achieved a detection limit of 10 copies/μL for all four target serogroups, amplification efficiencies of 89.64% to 100.42% (R^2^ ≥ 0.997), and no cross-reactivity with any of the negative controls, including SS4, APP, HPS, *Salmonella*, *E. coli*, and *S. aureus*. Intra-assay and inter-assay repeatability were below 3%. Testing 98 clinical porcine samples from Guangxi, China, yielded positivity rates of 26.53% for the serotype 1/14 group, 19.39% for the serotype 2/1/2 group, 17.34% for serotype 7, and 29.59% for serotype 9, respectively, with mixed-target detection in 30.61% of samples. This assay enables rapid, reliable detection of major zoonotic *S. suis* serotypes in porcine samples, though it detects serotypes 1 and 14 as a group, and serotypes 2 and 1/2 as a group, due to capsular locus sequence similarity—a limitation acceptable for surveillance given the high pathogenicity within each group. This tool supports One Health-based veterinary surveillance for zoonotic disease control.

## Simple Summary

*Streptococcus suis* is a dangerous bacterium that can infect both pigs and humans, causing diseases such as meningitis and septic shock. Currently, several tools are available for simultaneous detection of 1 to 3 serotypes of *Streptococcus suis*; however, to improve detection efficiency, we aimed to develop an assay capable of simultaneously detecting four serotypes. This study developed a new testing method that can simultaneously identify four major serotypes in a single test. The method shows high sensitivity, strong specificity, and stable results. In the 98 samples from sick pigs, all four serotypes were detected, with varying prevalence among them. This tool can help veterinarians and public health authorities track the spread of the bacterium more quickly, enabling timely measures to control outbreaks, thereby safeguarding livestock industry safety and public health.

## 1. Introduction

*Streptococcus suis* (*S. suis*, SS) is a Gram-positive bacterial pathogen that causes severe diseases in both pigs and humans, including meningitis, septic shock, endocarditis, and arthritis [1]. While *S. suis* is recognized as one of the most important bacterial pathogens affecting the swine industry worldwide, its significance as a zoonotic agent has received increasing attention over the past two decades [2,3]. In Southeast Asia, *S. suis* has become a leading cause of adult bacterial meningitis, with mortality rates reaching 10–20% even under appropriate antibiotic treatment [4,5].

The capsule polysaccharide (CPS) is the major virulence determinant of *S. suis* and forms the basis for serological classification [6]. To date, 29 serotypes (1–19, 21, 23–25, 27–31, 1/2) have been identified according to the antigenic differences in CPS [7]. The virulence potential, however, differs considerably among these serotypes, largely due to genetic variations in the *cps* gene cluster, which determine the biosynthesis and structure of CPS and consequently affect host immune recognition and bacterial survival [8,9]. Among these, serotypes 1, 2, 7, and 9 are most frequently associated with clinical disease in both pigs and humans across different geographical regions [10], with serotypes 2, 7, and 9 being particularly well-documented in recent studies from China [11]. Importantly, these serotypes exhibit marked differences in virulence and epidemiological patterns. Serotype 2 is globally recognized as the most virulent and predominant zoonotic serotype [12]. Serotype 9 is highly prevalent in Europe and Asia, while serotypes 1 and 7, although considered moderately pathogenic, have shown rapidly increasing detection rates in Chinese pig farms in recent years [13,14]. Mixed-target detection with multiple serotypes, most frequently serotypes 2 and 7, or serotypes 2 and 9, are also common in pig herds, further complicating disease surveillance and control [9,15,16].

From a public health perspective, pigs serve as the primary reservoir and the main source of human *S. suis* infection [17,18]. Human cases occur predominantly through direct contact with infected pigs or contaminated pork products, placing occupationally exposed populations, such as farmers, slaughterhouse workers, and veterinarians, at highest risk [19,20]. However, the zoonotic risk is not uniform across all *S. suis* strains, as different serotypes and genotypes vary considerably in their ability to cause human disease [21,22]. Therefore, surveillance of *S. suis* in swine populations is not merely a veterinary task but a public health necessity [23]. By tracking the distribution and emergence of specific serotypes in pigs, public health authorities can detect the introduction or re-emergence of highly pathogenic serotypes before human cases appear, provide early warnings to high-risk occupational groups, and inform the development of region-specific prevention strategies [24]. Rapid and accurate serotyping of *S. suis* from porcine samples enables early detection of emerging serotypes, assessment of zoonotic risk, and implementation of targeted interventions [25]. Conversely, the absence of systematic surveillance in pigs may delay the recognition of emerging zoonotic serotypes, potentially leading to unrecognized human outbreaks and missed opportunities for intervention [26].

Traditional serotyping methods based on agglutination are labor-intensive, time-consuming, and limited by the availability of high-quality antisera. Conventional PCR assays offer improved specificity but suffer from limited throughput and quantification capability, and require post-amplification electrophoresis, which extends the time to result and increases the risk of cross-contamination [27]. Real-time quantitative PCR (qPCR) overcomes these limitations by enabling closed-tube amplification with real-time fluorescence monitoring, shorter turnaround time, and higher sample throughput [28]. Several multiplex qPCR assays have been reported for the detection of specific *S. suis* serotypes [11,29], such as serotypes 2, 7, and 9 [30,31,32]. However, a four-plex assay covering serotypes 1, 2, 7, and 9, which together account for the majority of zoonotic infections in Asia, remains unavailable. Furthermore, existing methods are often reported as technical developments without explicit linkage to public health or surveillance applications.

To address this gap, we developed and evaluated a multiplex TaqMan qPCR assay targeting four serotype-specific CPS genes (*cps1I*, *cps2I*, *cps7L*, and *cps9J*) for the simultaneous detection of *S. suis* groups 1/14, 2/1/2, 7, and 9 in porcine samples. It is designed as a supplementary serogroup-specific screening assay for contexts where serotypes 1, 2, 7, and 9 are of primary epidemiological concern. While this approach does not detect all potentially pathogenic serotypes, it offers a rapid, cost-effective, and high-throughput method for serogroup surveillance. In addition to serotyping, pathotyping PCRs targeting virulence-associated genes such as *mrp*, *epf*, and *sly* have been developed for *S. suis* to assess strain-level pathogenic potential. While pathotyping provides complementary information on virulence, serotyping remains essential for tracking capsular type distribution and guiding vaccine development. Our assay focuses on the latter—serogroup-specific surveillance of the most prevalent zoonotic serotypes. We optimized the reaction conditions, assessed its analytical performance (sensitivity, specificity, and reproducibility), and validated its utility using clinical field samples from Guangxi, China. By enabling rapid serotype identification directly from porcine specimens, this assay provides a practical tool for veterinary surveillance and serves as an early-warning platform for zoonotic risk assessment within a One Health framework.

## 2. Materials and Methods

### 2.1. Bacteria and Clinical Samples

Bacterial strains of *S. suis* serotypes 2, *S. suis* serotypes 4, *S. suis* serotypes 9, *Actinobacillus pleuropneumoniae* (APP), *Haemophilus parasuis* (HPS), *Salmonella Typhimurium*, *Escherichia coli* (*E. coli*), and *Staphylococcus aureus* (*S. aureus*) were provided by Nanning Zhufulai Animal Health Management Co., Ltd. (Nanning, Guangxi, China). Serotypes 2 and 9 were used for positive plasmid reference samples construction. *S. suis* serotypes 4, APP, HPS, *Salmonella*, *E. coli*, and *S. aureus* were used as cross-reactivity controls. Due to limited availability, actual bacterial or nucleic acid samples of serotypes 1 and 7 could not be obtained. Recombinant plasmids for serotypes 1 (GenBank accession: JX986790.1) and 7 (KC537369.1) were synthesized by Sangon Biotech Co., Ltd. (Shanghai, China).

A total of 98 clinical samples, all obtained from tissues showing obvious pathological lesions (comprising pork, lung, gastrointestinal, and liver/spleen tissues) were collected from individual pigs across multiple farms in Guangxi, China, at four time points (October and December 2025, and March and June 2026). These samples were provided by Nanning Zhufulai Animal Health Management Co., Ltd., which had previously determined their *S. suis* infection status using a fluorescence-based quantitative assay targeting the universal GDH gene. According to the provider’s criteria, GDH positivity was defined as Ct ≤ 35, and samples with Ct values between 35 and 40 were considered suspicious. Among them, 34 were identified as *S. suis*-positive and 64 as negative.

### 2.2. Specific Probes and qPCR Primers

The capsular polysaccharide (CPS) gene sequences of *S. suis* serotypes 1, 2, 7, and 9 were retrieved from the NCBI database. Highly serotype-specific and conserved regions were identified using BLAST (BLAST: Basic Local Alignment Search Tool) and CDD (Home—Conserved Domains—NCBI) tools, leading to the selection of *cps1I*, *cps2I*, *cps7L*, and *cps9J* fragments as target sequences for primer and probe design.

Primers and probes were designed using Oligo7 software. Primer candidates with melting temperatures (Tm) ranging from 56 °C to 62 °C, GC content between 40% and 60%, and expected amplicon sizes between 80 bp and 200 bp. Probe sequences were selected from the inter-primer region, with Tm values 5–10 °C higher than those of the corresponding primers probe. Potential dimer formations were analyzed, and specificity was verified using the Blast tool.

The TaqMan probes were 5′-labeled with distinct fluorophores: FAM for *cps9J*, VIC for *cps2I*, Texas Red for *cps1I*, and Cy5 for *cps7L*. All primers and probes used in the study were custom synthesized by Zymetas Medtech Co., Ltd. (Shanghai, China) (Table 1).

### 2.3. DNA Extraction and Plasmid Standard Construction

DNA was extracted from *S. suis* serotypes 2 and 9 using the FastPure Bacteria DNA Isolation Mini Kit (Vazyme, Nanjing, China) following the manufacturer’s instructions. The target genes *cps2I* and *cps9J* were amplified by PCR using the primers listed in Table 1. The optimal reaction mixture contained 10 µL of 2 × Spark Taq PCR Master Mix (with dye), 3 µL of DNA template, 5 µL of nuclease-free water, and 0.5 pmol/µL of each primer. The amplification program was: 95 °C for 5 min; followed by 35 cycles of 98 °C for 5 s, 55 °C for 10 s, and 72 °C for 10 s.

The amplified fragments were cloned into the 5 min TA/Blunt-Zero Cloning Kit (Vazyme, Nanjing, China) to generate recombinant plasmids, which were then propagated in *E. coli* DH5α competent cells. Plasmids were validated by PCR and sequenced by Sangon Biotech Co., Ltd. Plasmid concentrations were determined using an L-SP-LB Ultramicro UV-Vis Spectrophotometer (LABGIC/Beijing Lanjieke Technology Co., Ltd., Beijing, China). Copy numbers were calculated using the following formula:

Copies/μL= (6.02 × 10^23^) × (X ng/μL × 10^−9^)/(plasmid length in bp × 660) (1)

where X is the plasmid concentration in ng/μL. The four recombinant plasmids were mixed at equal concentrations (1 × 10^9^ copies/μL each) and serially diluted 10-fold with DEPC-treated water to concentrations ranging from 10^8^ to 10^0^ copy/μL to serve as standards for subsequent experiments.

### 2.4. Optimization of qPCR Conditions

The optimization was performed using an equal mixture of four recombinant plasmid standards (*cps1I*, *cps2I*, *cps7L*, and *cps9J*) at a final concentration of 1 × 10^7^ copies/μL as the template. Nuclease-free water was used as the negative control. Annealing temperatures of 58 °C, 60 °C, 62 °C were tested, and 60 °C was determined to be optimal. Primer and probe concentrations were subsequently optimized at this annealing temperature. The final primer concentrations were 0.3 pmol/μL for *cps1I* and *cps7L*, and 0.1 pmol/μL for *cps2I* and *cps9J*. The optimal probe concentration was 0.1 pmol/μL for all four targets.

The final qPCR reaction mixture (20 μL) contained 10 μL of 2 × qPCR Probe DNA Master Mix, supplied by Zymetas Medtech Co., Ltd., 4 μL of DNA template, 4 μL of nuclease-free water, and the optimized primers and probes. The amplification program was: 95 °C for 5 min, followed by 40 cycles of 95 °C for 5 s and 60 °C for 30 s.

Each reaction was performed on a Roche LightCycler^®^ 96 (LC96) Real-Time PCR System (Roche Diagnostics GmbH, Basel, Switzerland). Data analysis was carried out using the Roche LightCycler^®^ 96 Software, version 1.1, and the exported data were plotted with Origin 2024. The baseline was set to the default value of 0.05, as defined by the LC96 software. Ct values were calculated automatically by the software as the crossing point where the fluorescence signal rises above the baseline noise band.

### 2.5. Evaluation of Primer–Probe Interactions and Multiplex qPCR Performance

To investigate the potential competitive interactions among primers and probes, as well as the formation of primer dimers in the multiplex reaction, each primer–probe pair was tested individually in single-plex qPCR assays and in combination in the multiplex qPCR format under optimal reaction conditions, using a template concentration of 10^3^ copies/μL. The performance was assessed by comparing mean Ct values obtained from the three independent replicates, together with fluorescence curve intensities, between the single-plex and multiplex formats to detect any interference.

### 2.6. Sensitivity Determination

Ten-fold serial dilutions of the recombinant plasmid standards, ranging from 1 × 10^7^ to 10^0^ copy/μL, were used as templates, nuclease-free water was used as the no-template control (NTC). Each dilution was tested in triplicate using the optimized multiplex TaqMan qPCR assay. The limit of detection (LOD) was defined as the lowest concentration at which detectable fluorescence signals were obtained in all three technical replicates from each of three independent experiments.

Standard curves were generated by plotting the log_10_-transformed copy numbers against the corresponding Ct values for each serotype. Linear regression equations and amplification efficiencies were calculated from the standard curves.

### 2.7. Specificity Analysis

To evaluate specificity, the four target plasmids (*cps1I*, *cps2I*, *cps7L*, and *cps9J*) at 1 × 10^7^ copies/μL were used as positive controls. DNA extracted from SS4, APP, HPS, *Salmonella*, *E. coli*, and *S. aureus* were used as negative controls to assess cross-reactivity, DNA was extracted using the FastPure Bacteria DNA Isolation Mini Kit (Vazyme) following the manufacturer’s instructions. Nuclease-free water was used as the NTC.

Specificity was confirmed if amplification curves were observed only in the positive controls and exclusively in their corresponding fluorescence channels, with no detectable signal from either the specificity controls or the NTC.

### 2.8. Precision Evaluation

Precision was evaluated using recombinant plasmid standards at three concentrations: 1 × 10^7^, 1 × 10^5^, and 1 × 10^3^ copies/μL. Intra-assay repeatability was assessed by testing three replicates within a single experiment, while inter-assay repeatability was assessed across three independent experiments.

The coefficient of variation (CV) was calculated for each group using the formula:

CV = (SD/Mean) × 100%(2)

where SD is the standard deviation and mean is the average Ct value of the replicates. A CV below 3% was considered acceptable.

### 2.9. Clinical Sample Testing Using Multiplex TaqMan qPCR and Conventional PCR

A total of 98 clinical samples were analyzed. From each sample, approximately 2 mg of tissue was homogenized. DNA was extracted using the FastPure Bacteria DNA Isolation Mini Kit (Vazyme) following the manufacturer’s instructions.

All samples were tested in parallel using the multiplex TaqMan qPCR assay developed in this study and the multiplex PCR method described by Liu et al. [27] for *S. suis* serotyping. For the qPCR assay, a sample was considered positive for a given serotype if the amplification curve in the corresponding fluorescence channel had a Ct value < 38. For the conventional multiplex PCR, a sample was considered positive for a given serotype if a band of the expected size [27] was clearly visible after agarose gel electrophoresis. Results from the two methods were compared to assess the accuracy of the qPCR assay.

## 3. Results

### 3.1. Plasmid Standard Verification

**Figure 1:**
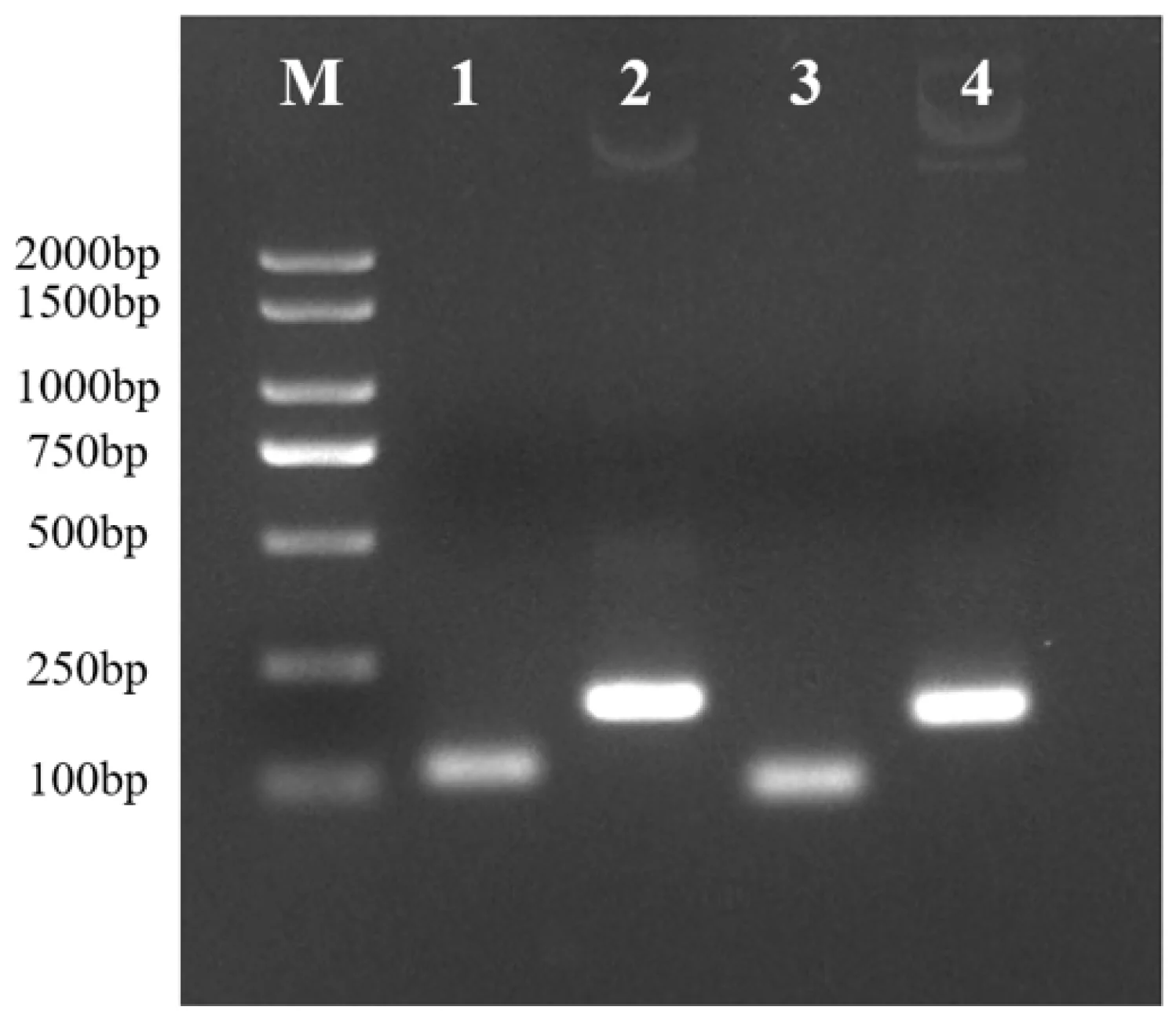
Gel electrophoresis results. M: 2000 bp DNA Marker; 1–4: *cps1I*, *cps2I*, *cps7L* and *cps9J*.

**Figure 2:**
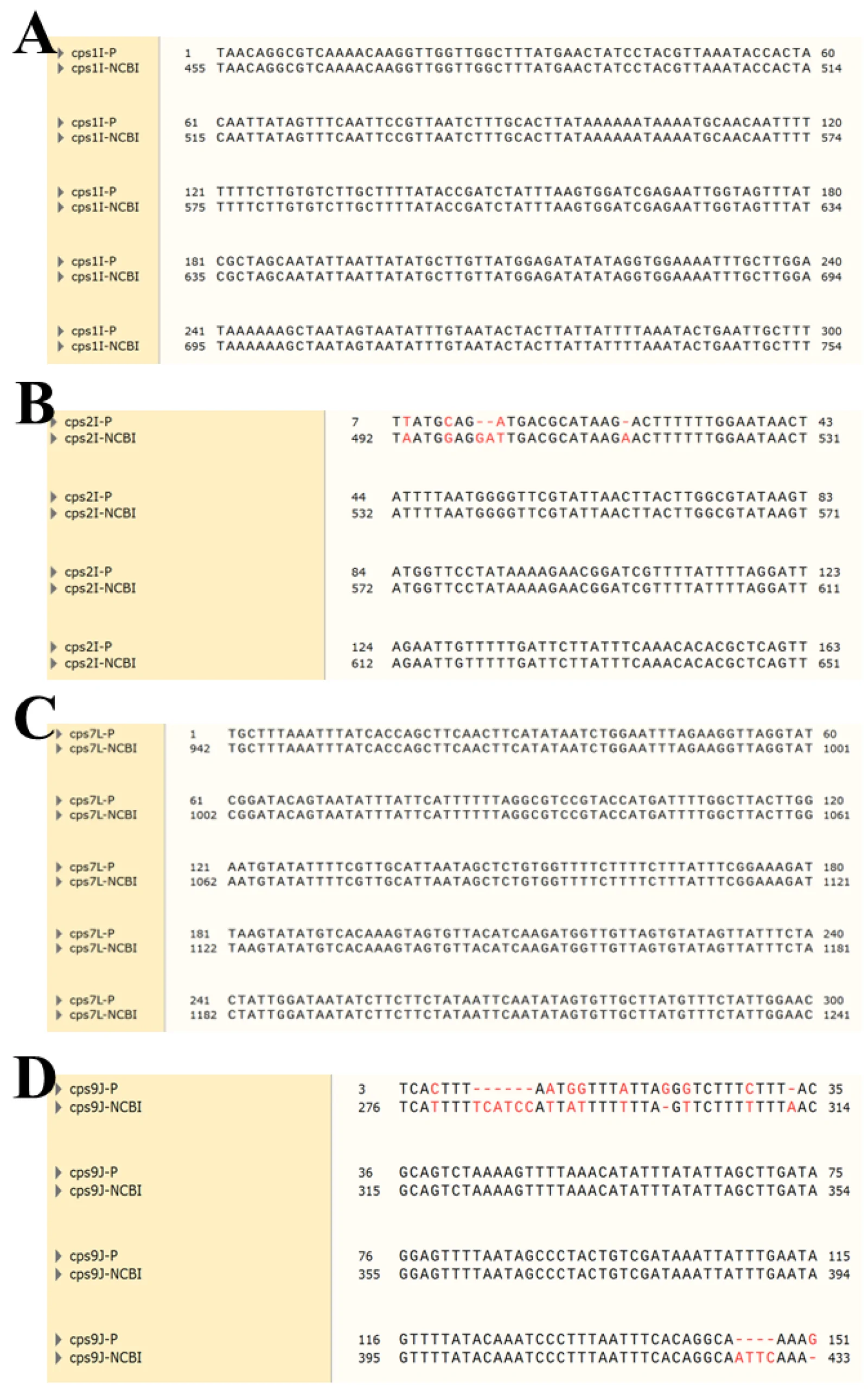
BLAST alignment results of recombinant plasmid sequences. (**A**) *cps1I* BLAST results; (**B**) *cps2I* BLAST results; (**C**) *cps7L* BLAST results; (**D**) *cps9J* BLAST results.

The constructed recombinant plasmids were used as templates for PCR amplification with the primers listed in Table 1. The PCR products were analyzed by agarose gel electrophoresis. As shown in Figure 1, specific bands of the expected sizes were observed: 112 bp for *cps1I*, 195 bp for *cps2I*, 96 bp for *cps7L*, and 173 bp for *cps9J*, confirming successful plasmid construction. The plasmids were further validated by Sanger sequencing (Sangon Biotech Co., Ltd.). BLAST alignment of the sequencing results showed high sequence identity with the corresponding sequences in the NCBI database (Figure 2). Of these, (A) and (C) are artificially synthesized plasmids whose sequences are completely identical to the reference sequence (100% identity). (B) and (D) are plasmids constructed in this study using DNA templates extracted from clinically isolated *S. suis*, showing 99% and 96% sequence identity, respectively. The lower identity values resulted from base deletions caused by PCR amplification mismatches or plasmid contamination. These deletions are located in non-critical regions and have little impact on the research results.

### 3.2. Optimization of qPCR Conditions

**Figure 3:**
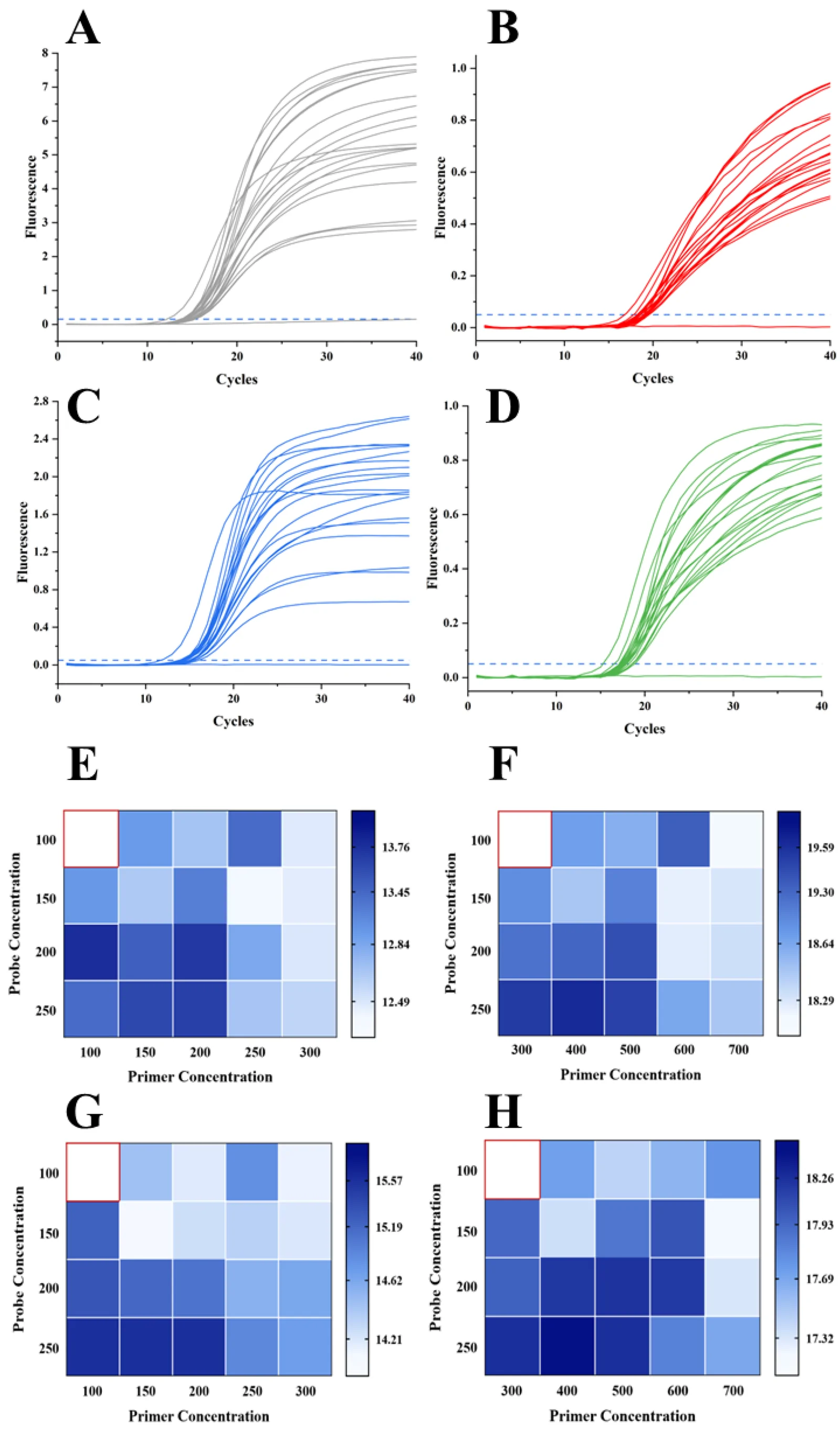
Primer and probe concentration optimization. (**A**) *cps1I* amplification curve; (**B**) *cps2I* amplification curve; (**C**) *cps7L* amplification curve; (**D**) *cps9J* amplification curve; (**E**) optimal primer and probe concentration for *cps1I*; (**F**) optimal primer and probe concentration for *cps2I*; (**G**) optimal primer and probe concentration for *cps7L*; (**H**) optimal primer and probe concentration for *cps9J*. Dashed lines in (**A**–**D**) represent the threshold, and red boxes in (**E**–**H**) indicate the concentration combinations with the lowest Ct values.

Optimal reaction conditions were determined by selecting the combination that produced the lowest Ct value and the highest fluorescence intensity. The optimal qPCR conditions were determined as follows: annealing temperature of 60 °C; The 20 µL reaction mixture now contains: 10 µL of 2× Probe qPCR Master Mix, 4 µL of template DNA, 0.3 µL of *cps1I* forward primer (20 µM working solution), 0.3 µL of *cps1I* reverse primer (20 µM working solution), 0.3 µL of *cps7L* forward primer (20 µM working solution), 0.3 µL of *cps7L* reverse primer (20 µM working solution), 0.1 µL of *cps2I* forward primer (20 µM working solution),0.1 µL of *cps2I* reverse primer (20 µM working solution), 0.1 µL of *cps9J* forward primer (20 µM working solution), 0.1 µL of *cps9J* reverse primer (20 µM working solution), 0.1 µL of each probe (20 µM working solution for all four probes), and 4 µL of nuclease-free water. The amplification program consisted of 95 °C for 5 min, followed by 40 cycles of 95 °C for 5 s and 60 °C for 30 s. The optimization results are presented in Figure 3.

### 3.3. Evaluation of Primer–Probe Interactions and Multiplex qPCR Performance

**Figure 4:**
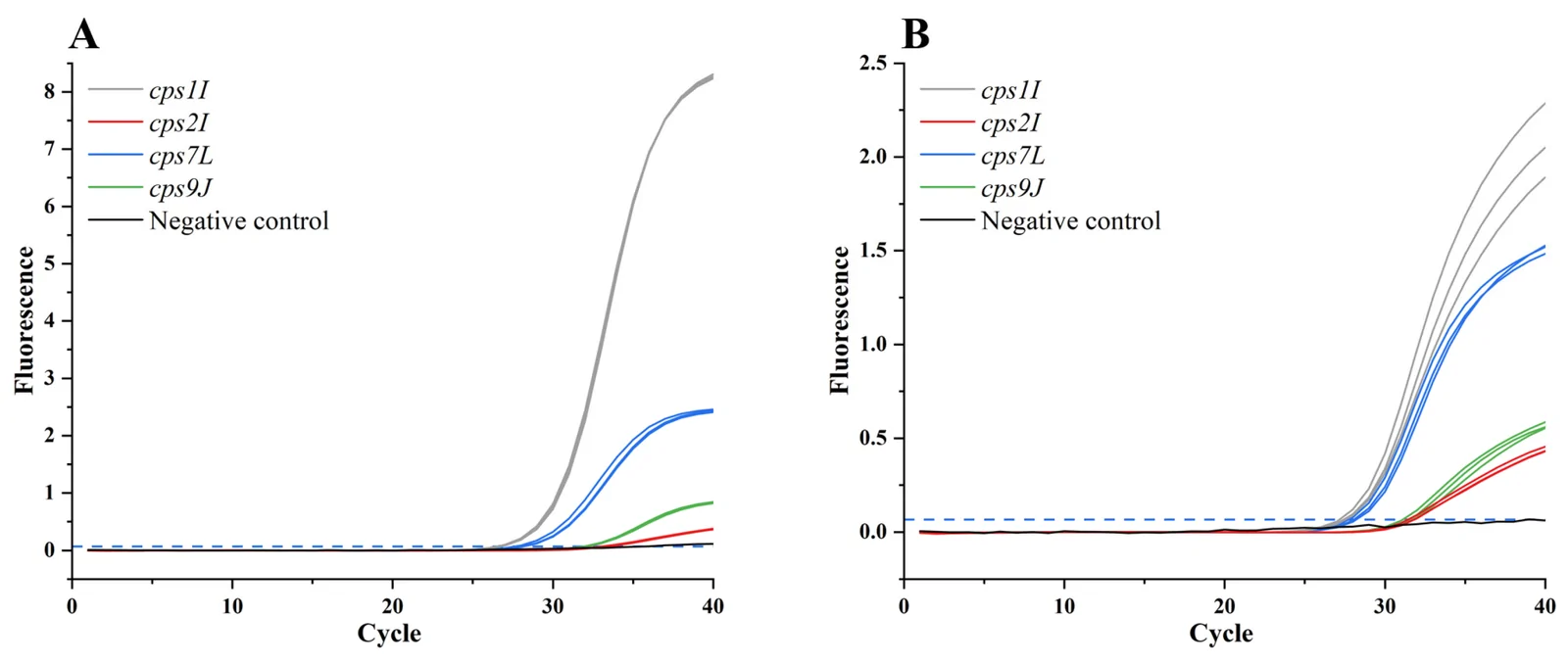
Primer and probe concentration optimization. (**A**) Single-plex qPCR results; (**B**) multiplex qPCR results. Dashed lines represent the threshold.

The results of single-plex and multiplex qPCR assays, each performed in triplicate using plasmid DNA at a concentration of 10^3^ copies/μL as the template, are presented in Figure 4. Even when detecting low-concentration samples, the multiplex reaction containing all four primer–probe pairs caused a modest decrease in fluorescence intensity relative to the single-plex reactions; however, the differences in Ct values between the two formats were consistently less than 1 cycle, and no fluorescence signal was observed in the negative controls. These findings collectively indicate that the competitive interference among primers and probes, including potential primer-dimer formation, remains within an acceptable range.

### 3.4. Sensitivity Determination

**Figure 5:**
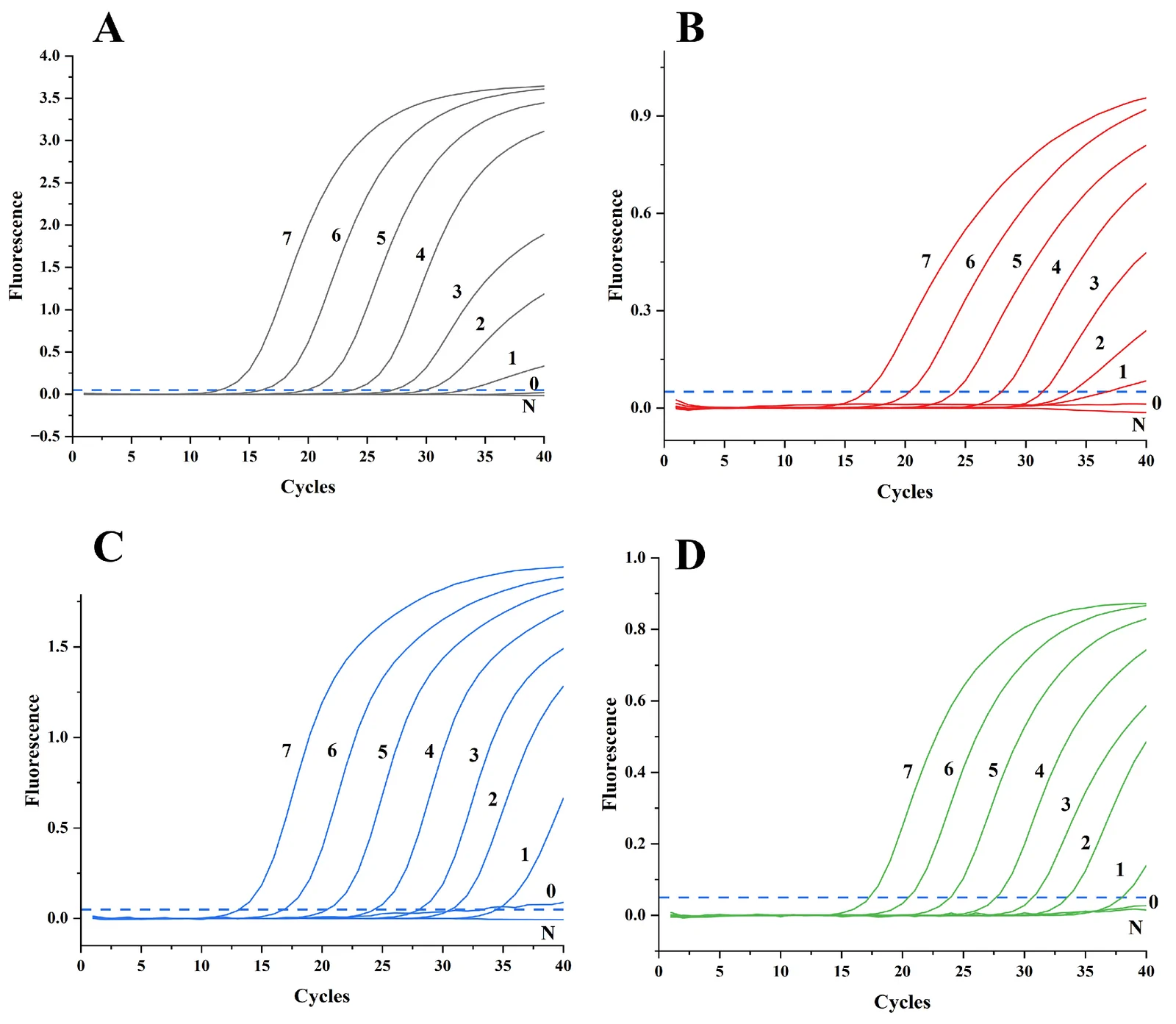
Amplification curves using about 10^0^–10^7^ copies/μL plasmids. (**A**) *cps1I* amplification curve, 0–7: 10^0^ to 10^7^ copies/μL; N: negative control; (**B**) *cps2I* amplification curve, 0–7: 10^0^ to 10^7^ copies/μL; N: negative control; (**C**) *cps7L* amplification curve, 0–7: 10^0^ to 10^7^ copies/μL; N: negative control; (**D**) *cps9J* amplification curve, 0–7: 10^0^ to 10^7^ copies/μL; N: negative control. Dashed lines represent the threshold.

The sensitivity of the multiplex qPCR assay was evaluated using ten-fold serial dilutions of recombinant plasmid standards ranging from 10^7^ to 10^0^ copy/μL. The amplification curves are shown in Figure 5. As presented in Figure S4, the limit of detection was determined to be 10^1^ copies/µL for all four serotypes, demonstrating high sensitivity of the established method.

Standard curves were generated by plotting the log_10_-transformed copy numbers against the corresponding Ct values. The linear regression equations and performance parameters were as follows: *cps1I*: y = −3.598x + 37.849, R^2^ = 0.998; *cps2I*: y = −3.378x + 40.901, R^2^ = 0.999; *cps7L*: y = −3.536x + 38.090, R^2^ = 0.997; and *cps9J*: y = −3.312x + 40.466, R^2^ = 0.997.

**Figure 6:**
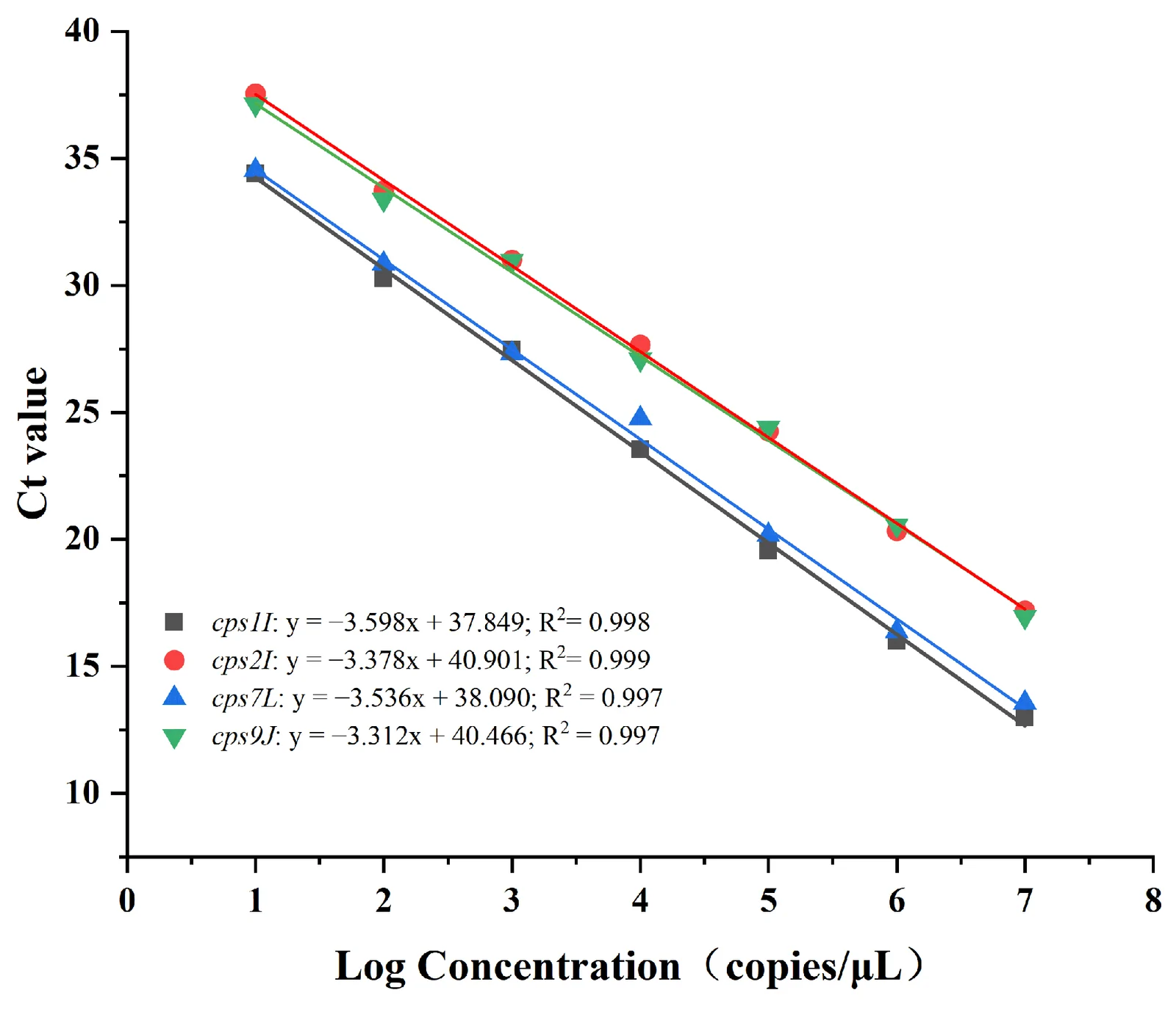
Standard curves using about 10^1^–10^7^ copies/μL plasmids.

Amplification efficiencies ranged from 89.64% to 100.42% (Figure 6). The high R^2^ values (all ≥ 0.997) indicate excellent linear relationships between plasmid copy numbers and Ct values, confirming the suitability of this assay for quantitative detection of *S. suis* in mixed samples.

### 3.5. Specificity Analysis

**Figure 7:**
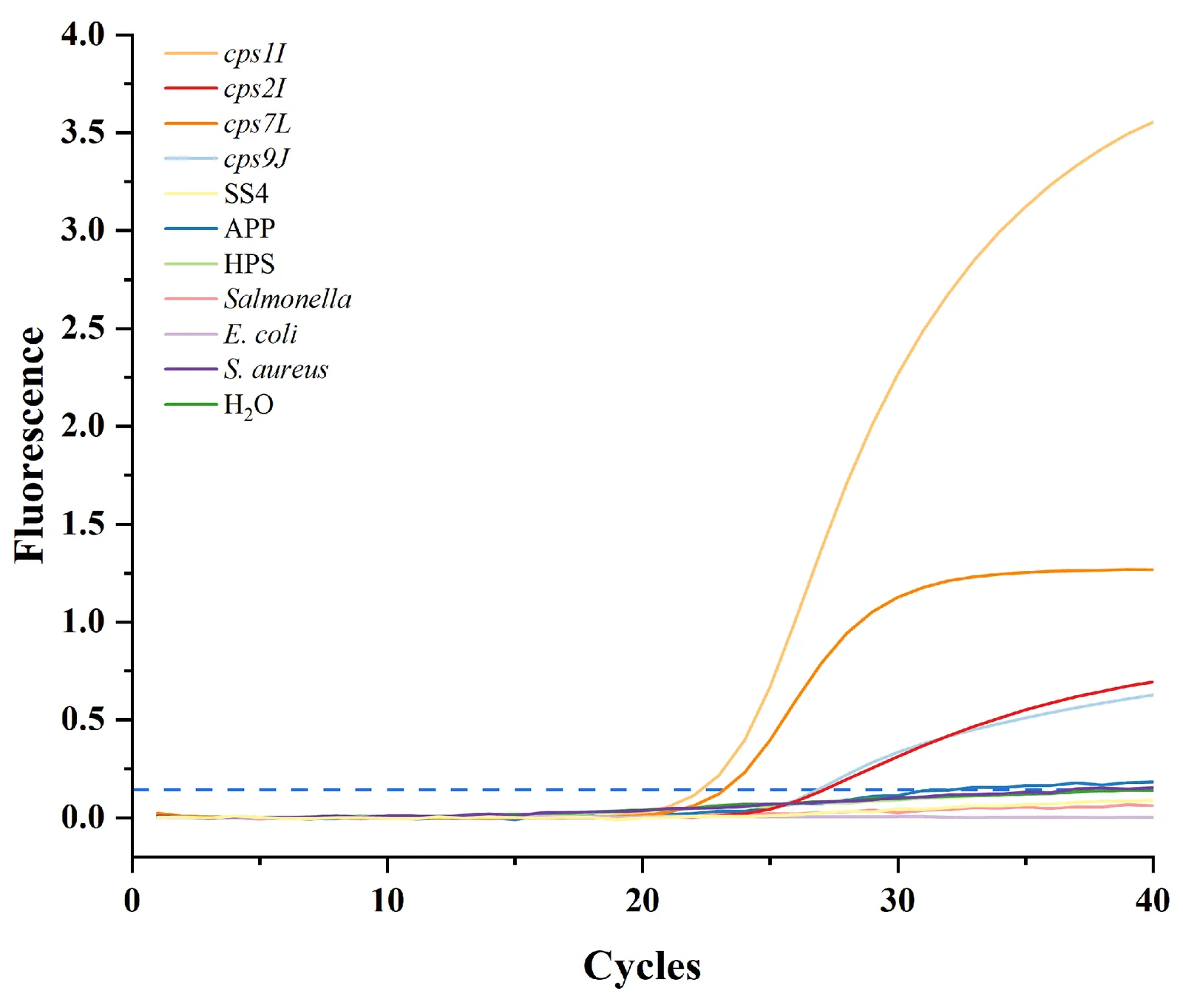
Specificity of the multiplex qPCR assay. Dashed lines represent the threshold.

Specificity was assessed using target plasmids (10^7^ copies/μL) as positive controls and DNA extracted from SS4, APP, HPS, *Salmonella*, *E. coli*, *S. aureus*, along with nuclease-free water, as negative controls. As shown in Figure 7, fluorescence signals were detected exclusively in the corresponding channels: Texas Red for *cps1I*, VIC for *cps2I*, Cy5 for *cps7L*, and FAM for *cps9J*. No amplification was observed from the non-target serotype controls or the no-template control, confirming high specificity and absence of cross-reactivity among the four target genes.

### 3.6. Precision Evaluation

Precision was evaluated at three plasmid concentrations: 10^7^, 10^5^, and 10^3^ copies/μL. Intra-assay repeatability was assessed from three replicates within a single experiment, and inter-assay repeatability was assessed from three independent experiments (Figure S1). The coefficients of variation (CV) for both intra- and inter-assay are summarized in Table 2. For *cps1I*, intra-assay CVs ranged from 0.36% to 1.08%, and inter-assay CVs from 1.64% to 2.44%. For *cps2I*, intra-assay CVs ranged from 0.45% to 0.80%, and inter-assay CVs from 0.20% to 1.11%. For *cps7L*, intra-assay CVs ranged from 0.60% to 1.09%, and inter-assay CVs from 0.90% to 1.21%. For *cps9J*, intra-assay CVs ranged from 0.16% to 0.98%, and inter-assay CVs from 1.85% to 2.30%. All CV values were below 3%, indicating excellent repeatability of the multiplex qPCR assay.

### 3.7. Clinical Sample Testing

A total of 98 clinical samples were tested using the established multiplex qPCR assay. Positivity was defined as Ct < 38 in the corresponding fluorescence channel and further categorized into three different pathogen loaded levels according to the following Ct value criteria: Ct < 16 indicated an extremely high pathogen load, 16 ≤ Ct ≤ 25 indicated a high pathogen load, 26 ≤ Ct ≤ 38 indicated a low pathogen load. For samples with mixed-target detection (two or more serotypes detected simultaneously), the lowest Ct value among all positive targets was used for sample-level categorization, as it represents the highest pathogen load within the sample and determines the detectability by conventional PCR. Among the 98 samples, 9 (9.18%) showed Ct values between 16 and 25 (high pathogen load), 31 (31.63%) showed Ct values between 26 and 38 (low pathogen load), and the remaining 58 (59.18%) were negative (Ct > 38). In total, 40 samples (40.82%) were identified as positive by the qPCR assay (Table S1 and Table 3).

The positivity rates were 26.53% (26/98) for serotype 1/14 group, 19.39% (19/98) for serotype 2/1/2 group, 17.34% (17/98) for serotype 7, and 29.59% (29/98) for serotype 9 (Figure S2 and Table S1). Mixed-target detection (detection of two or more serotypes in a single sample) was observed in 30 samples (30.61%).

Comparison of the qPCR assay with the GDH-based screening assay (performed by Nanning Zhufulai Animal Health Management Co., Ltd.) showed that 34 samples were identified as positive by the GDH assay, whereas 40 samples were positive by the qPCR assay (Table 3). Among the 40 qPCR-positive samples, six samples (No. 5, 10, 12, 17, 20, and 29) were negative by the GDH assay. In addition, the qPCR assay produced generally higher Ct values than the GDH assay for the same positive samples.

Comparison with the conventional multiplex PCR method [27] showed that the qPCR assay detected positive samples in all instances where PCR produced visible bands. However, the qPCR assay also identified additional positive samples that were negative by conventional PCR, consistent with the higher analytical sensitivity of qPCR (detection limit of 10 copies/μL versus 10^4^–10^5^ CFU for conventional PCR) (Table 4 and Figure S3). Discordant results between qPCR and conventional PCR were also observed in six samples (No. 5, 10, 12, 17, 20, and 29) (Table 4 and Table S1).

## 4. Discussion

Effective surveillance and control of zoonotic *S. suis* require tools capable of identifying serotypes with the highest epidemiological and clinical relevance [33]. The *cps* locus is not merely a taxonomic marker; it is intrinsically linked to the pathogenic potential of *S. suis*, as certain serotypes are rarely associated with clinical disease, while others are consistently recovered from invasive infections in both pigs and humans [34,35]. In Southeast Asia and several other regions, serotypes 1, 2, 7, and 9 are among the most frequently reported from clinical cases and are therefore considered priority targets for surveillance [36]. However, the distribution of pathogenic serotypes varies geographically, and serotypes not included in this study—such as 3, 4, 5, 8, and 16—have also been associated with clinical disease in certain regions or under specific epidemiological conditions [37]. Our assay is therefore designed as a serogroup-specific screening tool for contexts where serotypes 1, 2, 7, and 9 are of primary concern, rather than a universal serotyping method covering all known serotypes. In this study, a multiplex TaqMan qPCR assay was developed and validated for the simultaneous detection and quantification of *S. suis* serotypes 1, 2, 7, and 9 in porcine samples. The assay demonstrated high sensitivity (LOD of 10 copies/μL for all four serotypes), excellent specificity (no cross-reactivity among targets or with non-target serotypes), and good reproducibility (CV < 3%). When applied to 98 clinical field samples, the assay revealed positivity rates ranging from 17.34% to 29.59% for the four serotypes and detected mixed-target detection in nearly one-third of the positive samples. These findings confirm the assay’s practical utility for serogroup-specific screening of porcine samples in regions where serotypes 1, 2, 7, and 9 are of primary concern. These serotypes, particularly type 2, are well-documented zoonotic pathogens capable of causing severe human infections [38,39]. However, as this study did not include human clinical specimens, its performance on human isolates remains to be validated. In addition, the assay does not detect serotypes other than 1, 2, 7, and 9, and negative results should not be interpreted as absence of other potentially zoonotic serotypes. Future studies incorporating broader strain collections and human samples are needed to further assess the assay’s utility across different hosts and regions.

In the clinical sample testing, discordant results between the GDH assay and the qPCR assay were observed in six samples (GDH-negative but qPCR-positive). This discrepancy can be explained by several factors. First, the two assays target fundamentally different genes: the GDH assay detects a housekeeping gene present in all *S. suis* serotypes, whereas our qPCR assay targets serotype-specific *cps* genes. Second, the higher analytical sensitivity of the qPCR assay (LOD of 10 copies/μL) may enable detection of low-copy-number targets that fall below the detection threshold of the GDH assay. Third, the qPCR assay produced generally higher Ct values than the GDH assay for the same positive samples, which may be partly attributable to competitive interference among the four primer-probe sets in the multiplex reaction (Section 2.5, Figure 4). However, the possibility of non-specific amplification cannot be completely excluded and requires further investigation through sequencing or culture-based confirmation.

### 4.1. Limitations and Future Directions

Some limitations of this study should be noted, as they affect the interpretation of the results and the scope of the assay’s applicability. Firstly, all clinical samples were sourced from farms in Guangxi, a single geographic region. Consequently, the strain panel and sample set may not fully represent the genetic and serotype diversity of *S. suis* strains circulating in other regions or under different epidemiological contexts. This restricts the ability to comprehensively assess assay sensitivity and specificity against geographically distinct isolates, including both the target serotypes and other potentially pathogenic serotypes not included in this study. Due to this geographical constraint, actual bacterial or nucleic acid samples of serotypes 1 and 7 could not be obtained; thus, recombinant plasmid standards were used instead. Although the assay detected positive signals for serotypes 1 and 7 in clinical specimens, viable strains could not be isolated from these frozen tissues for use as reference controls. While plasmids are suitable for analytical validation, testing with authentic clinical isolates of these serotypes is needed in future work. Secondly, all clinical samples tested were of porcine origin. Although pigs are the primary zoonotic reservoir, direct validation using human clinical specimens (e.g., cerebrospinal fluid or blood) would further support the assay’s public health utility.

In addition, as discussed above, discordant results between the qPCR and GDH assays were observed in six samples. The possibility of non-specific amplification cannot be completely excluded and requires further investigation through sequencing or culture-based confirmation.

Although the multiplex qPCR assay established in this study can detect serotypes 1/14, 2/1/2, 7, and 9 of *S. suis*, it is unable to differentiate between serotypes 1 and 14, or between serotypes 2 and 1/2, owing to the high sequence homology (>99%) and SNP present in the target CPS gene regions; this is also one of the limitations of this study [40]. To overcome this limitation, future studies could employ HRM analysis, which has been proven capable of discriminating alleles that differ by as little as a single nucleotide. By designing primers flanking the SNP sites and optimizing the ramping rate, HRM may offer a feasible approach to resolve these four serotypes [41]. However, a validated HRM protocol specifically tailored for this purpose has not yet been established, and its performance should be carefully evaluated in clinical samples before routine application.

Although aerosol contamination is a potential concern in highly sensitive qPCR assays, strict quality-control measures were implemented in the present study, including physical separation of pre-amplification and post-amplification areas and the inclusion of negative controls. No amplification was observed in the negative controls, indicating that no detectable contamination occurred under the experimental conditions and supporting the reliability of the clinical testing results [41,42].

Further validation of the limit of detection is also warranted. In the present study, inter-assay and intra-assay repeatability tests were performed at the lowest template concentration, which is commonly adopted during qPCR assay development and provides an initial assessment of analytical sensitivity [42]. However, increasing the number of replicates at low target concentrations would allow a more robust estimation of detection probability. In particular, testing approximately 20 replicates per concentration combined with probit regression analysis would enable estimation of the concentration associated with a 95% probability of detection. Such validation would provide a more rigorous assessment of the analytical sensitivity of the assay.

In addition, although the multiplex qPCR assay established in this study exhibited favorable specificity, repeatability, and sensitivity, its value for definitive diagnosis may need to be interpreted in conjunction with serological and culture-based results [43]. Serological and molecular typing target fundamentally different biological features of *S. suis*: serological methods detect the capsular polysaccharide (CPS) antigen expressed on the bacterial surface (phenotype), whereas our qPCR assay detects specific sequences within the *cps* gene cluster (genotype). As documented in the literature, genotype–phenotype discrepancies are not uncommon in *S. suis* and may arise from CPS expression variability, capsule loss during subculture, or antisera cross-reactivity [9,43,44]. Therefore, the qPCR assay developed in this study was validated against reference strains of known genotype and sequencing-confirmed recombinant plasmids, following standard practices for molecular assay development. Prospective studies incorporating parallel isolation, serotyping, and sequencing of a larger panel of clinical isolates would be valuable to further explore genotype–phenotype relationships among circulating strains, although such an analysis was beyond the scope of the present methodological study. To this end, prospective studies that incorporate parallel isolation and serotyping are warranted to formally validate the clinical concordance of this molecular test.

Furthermore, we acknowledge that the presence of mixed-target detection observed in this study was based on the simultaneous amplification of multiple targets within the same DNA extract. Definitive confirmation of co-infection by multiple strains would require single-colony isolation followed by serotyping or sequencing, which was not performed in the present study. Therefore, the term “mixed-target detection” rather than “mixed infection” is used throughout the manuscript to reflect this limitation.

Future work should also explore expanding the assay to include additional serotypes (e.g., serotypes 3 and 14) and adapting it for other sample types, such as environmental samples from swine farms, to enable non-invasive surveillance.

### 4.2. Comparison with Existing Methods

Several molecular assays have been developed for *S. suis* serotyping, but most are limited to one to three serotypes per reaction. Wang et al. reported a quadruplex TaqMan qPCR assay targeting *S. suis* generic and serotypes 2, 7, and 9, achieving a detection limit of 10^0^ copy/μL [11]. Our assay extends this panel by including the serotype 1/14 group, which has shown rapidly increasing detection rates in Chinese pig farms in recent years [14]. However, our detection limit (10 copies/μL) is higher than that reported by [11]. This likely reflects the increased complexity of accommodating four TaqMan probes in a single reaction, which may lead to undesirable interactions such as primer dimer formation and competition for reaction components (e.g., polymerase and dNTPs), consequently reducing amplification efficiency [44]. Nevertheless, the achieved sensitivity (10 copies/μL) remains sufficient for clinical and surveillance applications, as most infected animals carry bacterial loads substantially above this threshold.

Compared with conventional multiplex PCR [27], our qPCR assay demonstrated superior sensitivity. The detection limit of the conventional multiplex PCR method is 10^4^–10^5^ CFU, and the DNA concentration in many of our clinical samples fell below this threshold. Therefore, the absence of detectable bands by multiplex PCR does not imply inconsistency between the two methods; rather, it reflects the higher analytical sensitivity of qPCR. In clinical sample testing, all samples with Ct values below 25 were positive by qPCR; however, six samples (No. 5, 10, 12, 17, 20, and 29) were negative by conventional PCR, which may indicate either higher analytical sensitivity of the qPCR assay or possible non-specific amplification, whereas only a subset of samples with Ct values between 26 and 38 (low pathogen load) yielded detectable bands by conventional PCR, consistent with the theoretical detection limit of conventional PCR.

Serological serotyping of *S. suis* typically requires type-specific antisera for capsular reaction or agglutination tests, or commercial ELISA kits [45,46]. However, the commercially available ELISA kits for *S. suis* are largely limited to the detection of serotype 2 antibodies and therefore cannot cover the four serotypes targeted by the present assay [47]. Acquiring a full panel of type-specific antisera covering all relevant serotypes is costly and requires specialized sourcing, which was not feasible within the scope of this study. Furthermore, the clinical samples used in this study were tissue specimens rather than bacterial isolates or serum samples. Serological tests, whether based on agglutination or ELISA, strictly require pure bacterial cultures or serum specimens [48,49]. In the present case, the residual DNA from these samples was depleted after the qPCR runs, and viable bacteria could not be recovered from the long-term frozen tissues. Consequently, retrospective serological comparison was not technically possible. Future investigations into serotype identification of *S. suis* should endeavor to integrate serological assays with qPCR-based approaches and to evaluate their concordance, thereby providing a more comprehensive framework for serotype surveillance and diagnostic applications.

### 4.3. Public Health and Surveillance Implications

Pigs are the primary reservoir of *S. suis* and the main source of human infection [2]. Human cases occur predominantly through direct contact with infected pigs or contaminated pork products, placing occupationally exposed populations at highest risk [50]. Given that *S. suis* is endemic in most pig herds worldwide, surveillance efforts should not aim to eradicate the pathogen but rather to track changes in serotype distribution and detect the emergence of highly pathogenic or zoonotic strains [51,52,53]. Our assay provides a practical tool for such surveillance by enabling rapid, high-throughput serotype identification directly from porcine tissue samples. However, it is inherently limited to detecting only the four targeted serogroups (1, 2, 7, and 9), and negative results do not exclude the presence of other serotypes that may be zoonotically relevant in different geographic or epidemiological settings.

Mixed infections comprising serotypes 1, 2, 7, and/or 9 of *S. suis* were observed in 30.61% of the positive samples. These findings suggest potential serotype diversity, a possibility that warrants further verification through strain isolation [54]. Our assay’s ability to simultaneously detect four serotypes in a single reaction makes it particularly well-suited for monitoring such mixed-target detection, which would be labor-intensive and inefficient using serotype-specific single-plex assays. Despite the limitations discussed above, the assay fills a practical gap in serotype-specific screening for regions where these serotypes are of primary epidemiological concern, and provides a useful adjunct to existing diagnostic workflows.

## 5. Conclusions

In summary, this multiplex TaqMan qPCR assay offers a sensitive, specific, and reproducible tool for the simultaneous detection of *S. suis* serotypes groups (1/14, 2/1/2, 7, and 9) in porcine samples. The assay enables rapid differentiation of these four serogroups and detection of co-circulation of multiple targets within single samples, addressing an important gap in current serotype-specific surveillance capabilities. Given that pigs are the primary source of human infection, this tool can support one-health-based strategies for reducing zoonotic transmission risk and guiding targeted public health interventions.

## Figures and Tables

**Table 1:** Specific probes and qPCR primers.

Primers and Probes	Sequence (5′-3′)	Product Length (bp)	GenBank Accession
*cps1I*-FP	TGTCTTGCTTTTATACCGAT	112	JX986790.1
*cps1I*-RP	TCCAAGCAAATTTTCCACCT		
*cps1I*-Probe	Texas Red-AACTACCAATTCTCGATCCACT-BHQ2		
*cps2I*-FP	GGGCAGTAGAAGGTATCGGT	195	KC537364.1
*cps2I*-RP	AAACTGAGCGTGTGTTTGAA		
*cps2I*-Probe	VIC-AATGGAGGATTGACGCATAAGAACTTT-BHQ1		
*cps7L*-FP	TCACCAGCTTCAACTTCATA	96	KC537369.1
*cps7L*-RP	TCATGGTACGGACGCCTAAA		
*cps7L*-Probe	Cy5-TCTGGAATTTAGAAGGTTAGGTATCGGA-BHQ3		
*cps9J*-FP	AGTTATTGTAGCAACAACCCTTCA	173	KC537370.1
*cps9J*-RP	TTGCCTGTGAAATTAAAGGGA		
*cps9J*-Probe	FAM-AGCTTGATAGGAGTTTTAATAGCCCTACTGTCG-BHQ1		

**Table 2:** Precision analysis of the qPCR methods.

Pathogens	Standard (Copies/μL)	CT (X ± SD)		CT (X ± SD)	
		Intra-Assay	CV/%	Inter-Assay	CV/%
*cps1I*	10^7^	12.54 ± 0.14	1.08	12.40 ± 0.27	2.16
	10^5^	19.73 ± 0.07	0.36	19.43 ± 0.32	1.64
	10^3^	27.46 ± 0.27	0.99	26.90 ± 0.66	2.44
*cps2I*	10^7^	16.88 ± 0.13	0.80	16.98 ± 0.19	1.11
	10^5^	24.00 ± 0.11	0.45	24.15 ± 0.27	1.10
	10^3^	31.40 ± 0.22	0.70	31.33 ± 0.06	0.20
*cps7L*	10^7^	13.33 ± 0.15	1.09	13.40 ± 0.16	1.18
	10^5^	20.27 ± 0.12	0.60	20.33 ± 0.18	0.90
	10^3^	28.14 ± 0.25	0.87	27.78 ± 0.34	1.21
*cps9J*	10^7^	17.10 ± 0.164	0.96	16.88 ± 0.39	2.30
	10^5^	24.11 ± 0.08	0.16	23.81 ± 0.45	1.89
	10^3^	31.10 ± 0.303	0.98	30.74 ± 0.57	1.85

**Table 3:** Comparison of qPCR and GDH assay results.

		Multiplex TaqMan qPCR		Sum
		Positive	Negative	
GDH	Positive	34/98 (34.69%)	0/98 (0%)	34/98 (34.69%)
	Negative	6/98 (6.12%)	58/98 (59.18%)	64/98 (65.31%)
	Sum	40/98 (40.82%)	58/98 (59.18%)	98

**Table 4:** Comparison of qPCR and conventional multiplex PCR results.

		Multiplex PCR		Sum
		Positive	Negative	
Multiplex TaqMan qPCR	16 ≤ Ct ≤ 25	3/98 (3.06%)	6/98 (6.12%)	9/98 (9.18%)
	26 ≤ Ct ≤ 38	1/98 (1.02%)	30/98 (30.61%)	31/98 (31.63%)
	>38	0	58/98 (59.18%)	58/98 (59.18%)
**Sum**		4/98 (4.08%)	94/98 (95.92%)	98

Note: For samples with mixed-target detection, the lowest Ct value among all amplified targets was used to categorize the sample into the corresponding Ct range (16–25 or 26–38), as this value represents the highest pathogen load within the sample and determines the likelihood of detection by conventional PCR.

## Data Availability

The original contributions presented in this study are included in the article/Supplementary Materials. Further inquiries can be directed to the corresponding authors.

## Supplementary Materials

The following supporting information can be downloaded at https://www.mdpi.com/article/10.3390/vetsci13090884/s1, Figure S1: Repeatability test results; Figure S2: qPCR detection results for *S. suis* clinical samples; Figure S3: PCR detection results for *S. suis* clinical samples; Figure S4: Determination of the limit of detection (LOD); Table S1: Clinical sample test results; Table S2: BLAST results of primers.
