# Supplementary material for: A Multiplex TaqMan qPCR Assay for Rapid Detection of Streptococcus suis Serotypes 1, 2, 7, and 9 with Cross-Reactivity to Serotypes 14 and 1/2
Source: Vet Sci. 2026 Aug 29;13(9):884. doi: 10.3390/vetsci13090884 (PMC13612046; doi:10.3390/vetsci13090884)
Supplement: Supplementary file 1 [file vetsci-13-00884-s001.zip › vetsci-4472782-supplementary.pdf]

Supporting information:

A Multiplex TaqMan qPCR Assay for Rapid Detection of *Streptococcus suis* Serotypes 1, 2, 7, and 9 with Cross-Reactivity to Serotypes 14 and 1/2

Jingye Huang [1], Tingting Yue [1], Jianing Huang [1], Qiong Chen [2], Min Zheng [3], Meiling Yu [1], Mingsheng Jiang [1], Xiulin Li [1,*] and Hailan Chen [1,*]

[1] Guangxi Key Laboratory of Animal Breeding, Disease Control and Prevention, College of Animal Science and Technology, Guangxi University, Nanning 530004, China
[2] Nanning Zhufulai Animal Health Management Co., Ltd., Nanning 530006, China
[3] Guangxi Center for Animal Disease Control and Prevention, Nanning 530001, China
* Correspondence: xiulinli@hotmail.com (X.L.); hlchen319@163.com (H.C.)

Supporting Figures

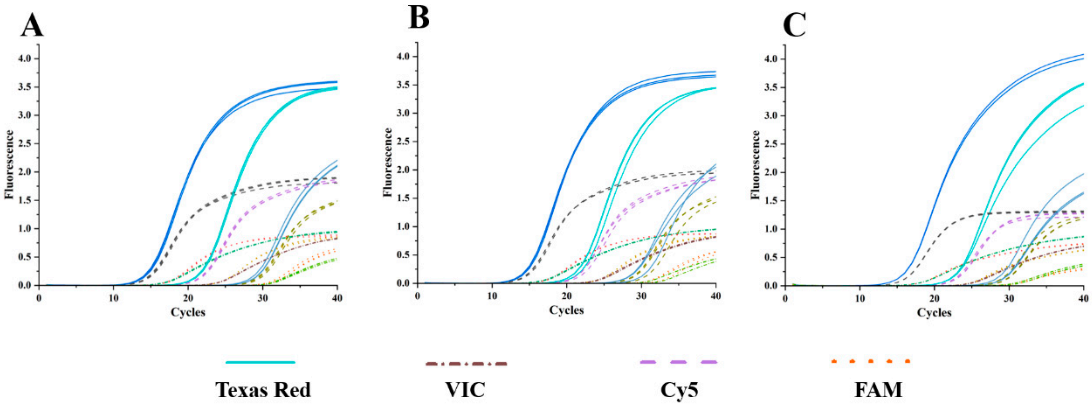

Figure S1. Repeatability test results. (**A**) intra-assay repeatability tests in January 2026; (**B**) intra-assay repeatability tests in February 2026; (**C**) intra-assay repeatability tests in March 2026.

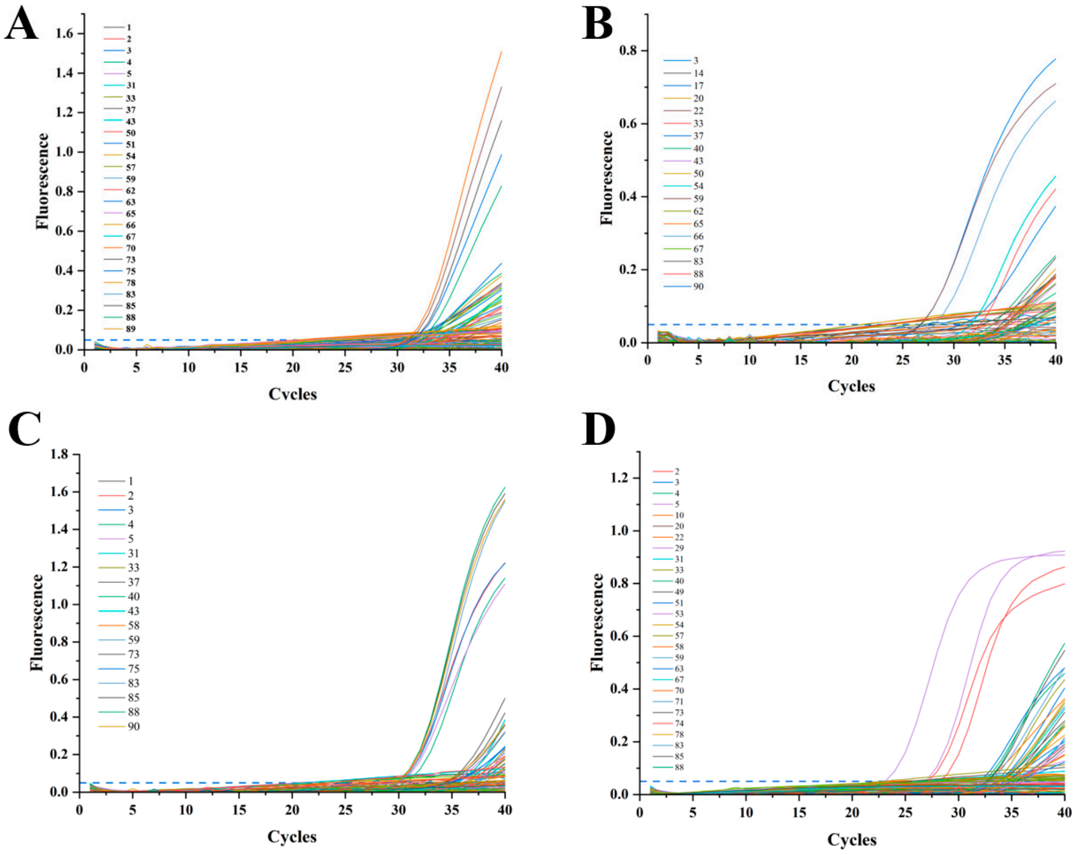

Figure S2. qPCR detection results for *S. suis* clinical samples. (**A**) Texas Red channel (*cps1I*); (**B**) VIC channel (*cps2I*); (**C**) Cy5 channel (*cps7L*); (**D**) FAM channel (*cps9J*). Dashed lines represent the threshold.

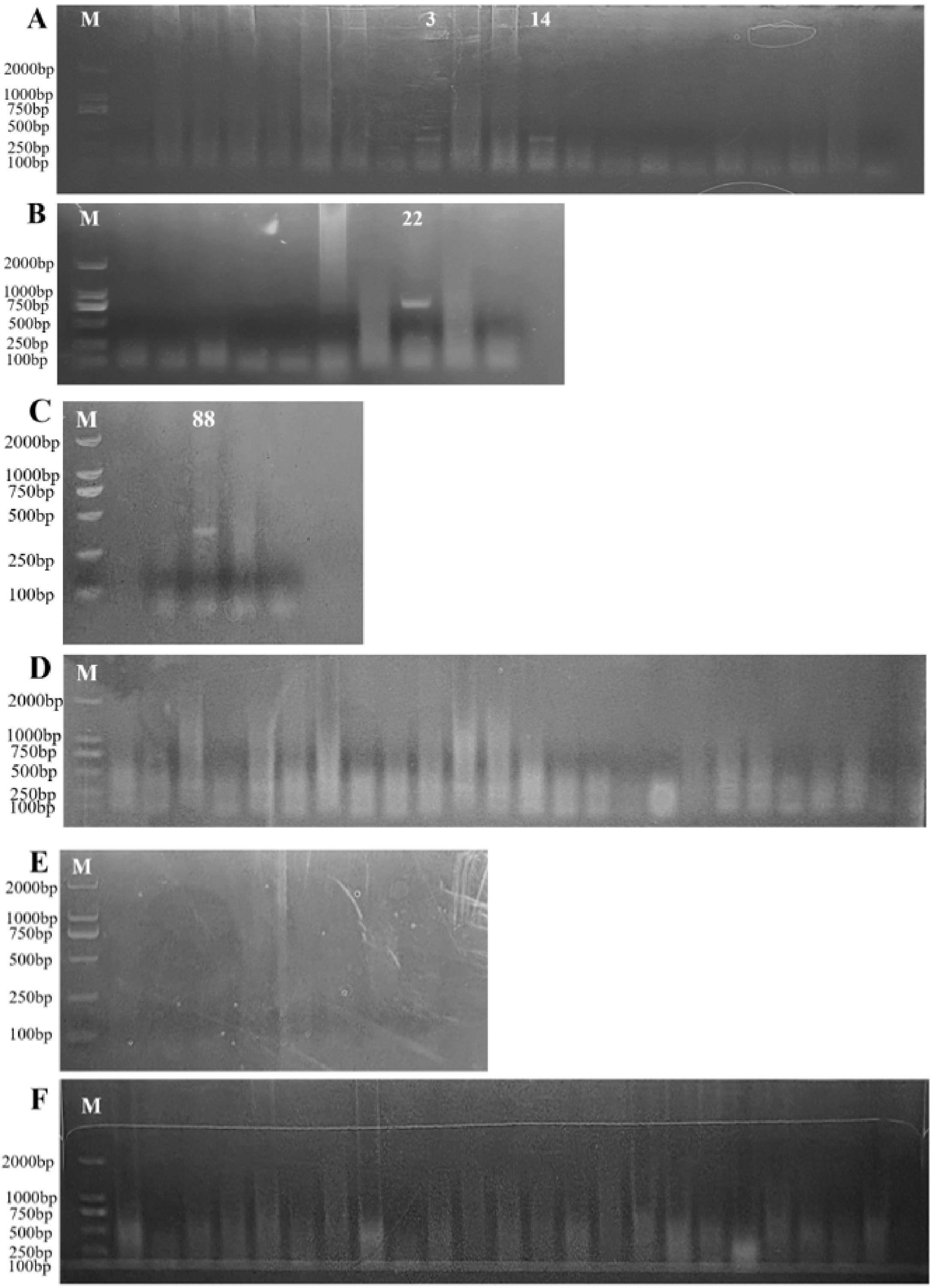

Figure S3. PCR detection results for *S. suis* clinical samples. M: 2000 bp DNA Marker. Samples with numbers have specific bands; samples without numbers are negative. (**A**) 3 and 14 correspond to the *cps2* gene; (**B**) 22 corresponds to the *cps7* gene; (**C**) 88 corresponds to the *cps2* gene; (**D**-**F**) No specific bands.

Table S1. Clinical sample test results.

Sample	Ct value					PCR result
	GDH	*cps1I*	*cps2I*	*cps7L*	*cps9J*	
1	31.33	No fluorescence	No fluorescence	34.37	No fluorescence	negative
2	31.21	33.56	No fluorescence	29.98	28.32	negative
3	27.11	34.02	24.89	30.00	32.01	*cps2*
4	31.21	32.25	No fluorescence	30.64	32.33	negative
5	31.21	33.95	No fluorescence	30.24	24.98	negative
6	No fluorescence	No fluorescence	No fluorescence	No fluorescence	No fluorescence	negative
7	No fluorescence	No fluorescence	No fluorescence	No fluorescence	No fluorescence	negative
8	No fluorescence	No fluorescence	No fluorescence	No fluorescence	No fluorescence	negative
9	No fluorescence	No fluorescence	No fluorescence	No fluorescence	No fluorescence	negative
10	22.25	No fluorescence	No fluorescence	No fluorescence	23.55	negative
11	No fluorescence	No fluorescence	No fluorescence	No fluorescence	No fluorescence	negative
12	22.25	No fluorescence	No fluorescence	No fluorescence	24.37	negative
13	No fluorescence	No fluorescence	No fluorescence	No fluorescence	No fluorescence	negative
14	27.11	No fluorescence	22.78	No fluorescence	No fluorescence	*cps2*
15	No fluorescence	No fluorescence	No fluorescence	No fluorescence	No fluorescence	negative
16	No fluorescence	No fluorescence	No fluorescence	No fluorescence	No fluorescence	negative
17	27.11	No fluorescence	23.16	No fluorescence	No fluorescence	negative
18	No fluorescence	No fluorescence	No fluorescence	No fluorescence	No fluorescence	negative
19	No fluorescence	No fluorescence	No fluorescence	No fluorescence	No fluorescence	negative
20	20.06	No fluorescence	21.20	No fluorescence	24.99	negative

21	No fluorescence	No fluorescence	No fluorescence	No fluorescence	No fluorescence	negative
22	31.33	31.49	34.96	29.70	32.65	*cps7*
23	No fluorescence	No fluorescence	No fluorescence	No fluorescence	No fluorescence	negative
24	No fluorescence	No fluorescence	No fluorescence	No fluorescence	No fluorescence	negative
25	No fluorescence	No fluorescence	No fluorescence	No fluorescence	No fluorescence	negative
26	No fluorescence	No fluorescence	No fluorescence	No fluorescence	No fluorescence	negative
27	No fluorescence	No fluorescence	No fluorescence	No fluorescence	No fluorescence	negative
28	No fluorescence	No fluorescence	No fluorescence	No fluorescence	No fluorescence	negative
29	22.25	No fluorescence	No fluorescence	No fluorescence	23.15	negative
30	No fluorescence	No fluorescence	No fluorescence	No fluorescence	No fluorescence	negative
31	No fluorescence	34.13	No fluorescence	34.86	35.00	negative
32	No fluorescence	No fluorescence	No fluorescence	No fluorescence	No fluorescence	negative
33	No fluorescence	34.45	34.63	34.97	33.55	negative
34	No fluorescence	No fluorescence	No fluorescence	No fluorescence	No fluorescence	negative
35	No fluorescence	No fluorescence	No fluorescence	No fluorescence	No fluorescence	negative
36	No fluorescence	No fluorescence	No fluorescence	No fluorescence	No fluorescence	negative
37	No fluorescence	34.21	32.60	34.88	No fluorescence	negative
38	No fluorescence	No fluorescence	No fluorescence	No fluorescence	No fluorescence	negative
39	No fluorescence	No fluorescence	No fluorescence	No fluorescence	No fluorescence	negative
40	31.33	No fluorescence	33.96	34.95	34.84	negative

Table S1. Clinical sample test results

Sample	Ct value					PCR result
	GDH	*cps1I*	*cps2I*	*cps7L*	*cps9J*	
41	No fluorescence	No fluorescence	No fluorescence	No fluorescence	No fluorescence	negative
42	No fluorescence	No fluorescence	No fluorescence	No fluorescence	No fluorescence	negative
43	33.37	32.17	34.87	34.54	No fluorescence	negative
44	No fluorescence	No fluorescence	No fluorescence	No fluorescence	No fluorescence	negative
45	No fluorescence	No fluorescence	No fluorescence	No fluorescence	No fluorescence	negative
46	No fluorescence	No fluorescence	No fluorescence	No fluorescence	No fluorescence	negative
47	No fluorescence	No fluorescence	No fluorescence	No fluorescence	No fluorescence	negative
48	No fluorescence	No fluorescence	No fluorescence	No fluorescence	No fluorescence	negative
49	31.33	No fluorescence	No fluorescence	No fluorescence	34.59	negative
50	33.37	33.02	33.79	No fluorescence	No fluorescence	negative
51	22.25	31.83	No fluorescence	No fluorescence	33.74	negative
52	No fluorescence	No fluorescence	No fluorescence	No fluorescence	No fluorescence	negative
53	22.25	No fluorescence	No fluorescence	No fluorescence	34.06	negative
54	31.05	29.50	26.71	No fluorescence	33.87	negative
55	No fluorescence	No fluorescence	No fluorescence	No fluorescence	No fluorescence	negative
56	No fluorescence	No fluorescence	No fluorescence	No fluorescence	No fluorescence	negative
57	31.21	30.85	No fluorescence	No fluorescence	34.01	negative
58	31.21	No fluorescence	No fluorescence	33.39	32.46	negative
59	No fluorescence	30.05	32.77	33.83	33.75	negative

60	No fluorescence	No fluorescence	No fluorescence	No fluorescence	No fluorescence	negative
61	No fluorescence	No fluorescence	No fluorescence	No fluorescence	No fluorescence	negative
62	27.11	31.84	30.64	No fluorescence	No fluorescence	negative
63	27.11	33.43	No fluorescence	No fluorescence	34.62	negative
64	No fluorescence	No fluorescence	No fluorescence	No fluorescence	No fluorescence	negative
65	27.11	33.93	29.68	No fluorescence	No fluorescence	negative
66	27.11	30.66	27.09	No fluorescence	No fluorescence	negative
67	20.06	30.46	33.88	No fluorescence	33.87	negative
68	No fluorescence	No fluorescence	No fluorescence	No fluorescence	No fluorescence	negative
69	No fluorescence	No fluorescence	No fluorescence	No fluorescence	No fluorescence	negative
70	No fluorescence	33.15	No fluorescence	No fluorescence	34.67	negative
71	31.05	No fluorescence	No fluorescence	No fluorescence	34.74	negative
72	No fluorescence	No fluorescence	No fluorescence	No fluorescence	No fluorescence	negative
73	31.33	29.71	No fluorescence	34.36	33.80	negative
74	31.33	No fluorescence	No fluorescence	No fluorescence	27.17	negative
75	31.05	32.03	No fluorescence	34.56	No fluorescence	negative
76	No fluorescence	No fluorescence	No fluorescence	No fluorescence	No fluorescence	negative
77	No fluorescence	No fluorescence	No fluorescence	No fluorescence	No fluorescence	negative
78	No fluorescence	32.47	No fluorescence	No fluorescence	34.73	negative
79	No fluorescence	No fluorescence	No fluorescence	No fluorescence	No fluorescence	negative
80	No fluorescence	No fluorescence	No fluorescence	No fluorescence	No fluorescence	negative
81	No fluorescence	No fluorescence	No fluorescence	No fluorescence	No fluorescence	negative

Table S1. Clinical sample test results.

Sample	Ct value					PCR result
	GDH	*cps1I*	*cps2I*	*cps7L*	*cps9J*	
82	No fluorescence	No fluorescence	No fluorescence	No fluorescence	No fluorescence	negative
83	22.25	31.10	34.93	29.95	32.98	negative
84	No fluorescence	No fluorescence	No fluorescence	No fluorescence	No fluorescence	negative
85	34.05	30.84	No fluorescence	29.76	32.74	negative
86	No fluorescence	No fluorescence	No fluorescence	No fluorescence	No fluorescence	negative
87	No fluorescence	No fluorescence	No fluorescence	No fluorescence	No fluorescence	negative
88	20.90	No fluorescence	20.96	No fluorescence	24.32	*cps2*
89	No fluorescence	No fluorescence	No fluorescence	No fluorescence	No fluorescence	negative
90	33.37	31.50	33.94	No fluorescence	No fluorescence	negative
91	No fluorescence	No fluorescence	No fluorescence	No fluorescence	No fluorescence	negative
92	No fluorescence	No fluorescence	No fluorescence	No fluorescence	No fluorescence	negative
93	No fluorescence	No fluorescence	No fluorescence	No fluorescence	No fluorescence	negative
94	No fluorescence	No fluorescence	No fluorescence	No fluorescence	No fluorescence	negative
95	No fluorescence	No fluorescence	No fluorescence	No fluorescence	No fluorescence	negative
96	No fluorescence	No fluorescence	No fluorescence	No fluorescence	No fluorescence	negative
97	No fluorescence	No fluorescence	No fluorescence	No fluorescence	No fluorescence	negative
98	No fluorescence	No fluorescence	No fluorescence	No fluorescence	No fluorescence	negative

Table S2. BLAST results of primers

Primers	Expected amplicon sequences	Products on target templates
cps1I	TGTCTTGCTTTTATACCGAT CTATTTAAGTGGATCGAGAA TTGGTAGTTTATCGCTAGCA ATATTAATTATATGCTTGTT ATGGAGATATATAGGTGGAA AATTTGCTTGGA	CP109942.1, CP109941.1, CP085088.1, CP085085.1, LR594043.1, JX986790.1, AB737817.1, CP002651.1, JF273644.1, CP002465.1, AF155804.1, CP152119.1, CP139875.1, CP134477.1, AB737822.1, CP139880.1, JF273653.1, JX986804.1
cps2I	GGGCAGTAGAAGGTATCGGT TTTAGTCAGGGTTTTAATGG AGGATTGACGCATAAGAACT TTTTTGGAATAACTATTTTA ATGGGGTTCGTATTAACTTA CTTGGCGTATAAGTATGGTT CCTATAAAAGAACGGATCGT TTTATTTTAGGATTAGAAT TGTTTTTGATTCTTATTTCA AACACACGCTCAGTTT	CP181158.1, CP181191.1, CP181205.1, CP142676.1, CP139881.1, OX352944.1, OX352941.1, CP139879.1, OX352831.1, CP102154.1, CP100326.1, CP100332.1, CP100334.1, CP095463.1, CP082197.1, CP082199.1, CP047248.1, CP058742.1, CP031379.1, CP030023.1, CP065431.1, CP100338.1, CP030020.1, CP031377.1, OX352996.1, CP030017.1, CP100336.1, CP102137.1, CP102140.1, CP102143.1, CP100330.1, CP102746.1, OX352940.1, CP082200.1, CP091422.1, CP091423.1, CP082948.1, LR738722.1, LR738721.1, LR738720.1, CP141904.1, CP100328.1, CP095182.1, CP025419.1, CP100432.1, LS483418.1, CP024126.1, CP100340.1, CP024050.1, CP121670.1, CP016175.1, CP102748.1, CP020863.1, CP082201.1, KX870076.1, CP100418.1, KX870052.1, CP003736.1, DQ410849.1, CP181156.1, DQ256457.1, AF118389.1, CP000837.1
cps7L	TCACCAGCTTCAACTTCAT ATAATCTGGAATTTAGAAGG TTAGGTATCGGATACAGTAA TATTTATTCATTTTTTAGGC GTCCGTACCATGA	CP002641.1, BR001004.1, KC537369.1, CP058741.1, CP071806.1, CP100431.1, CP139882.1, PV258565.1
cps9J	AGTTATTGTAGCAACAACCC TTCATTTTTCATCCATTAT TTTTTTAGTTCTTTTTTTAA CGCAGTCTAAAAGTTTTAAA CATATTTATATTAGCTTGATA GGAGTTTTAATAGCCCTACT GTCGATAAATTATTTGAATA GTTTTATACAAATCCCTTTA ATTTCACAGGCAA	CP102138.1, CP082203.1, CP058740.1, LR738724.1, KY574603.1, KC537370.1, BR001006.1, AB738321.1, JF273651.1

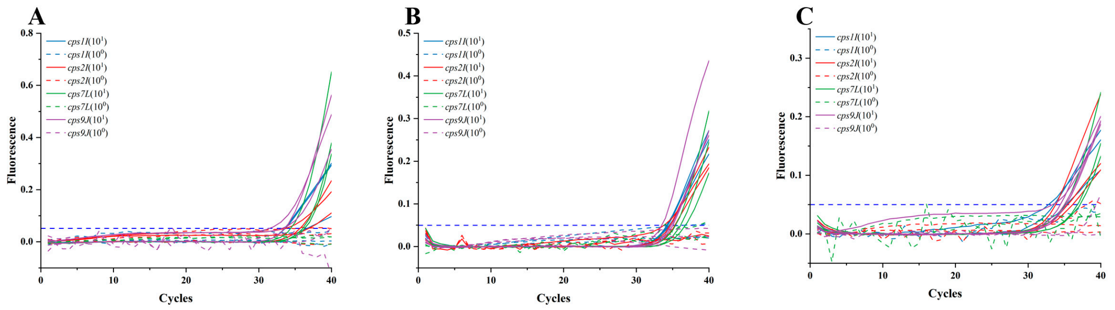

Figure S4. Determination of the limit of detection (LOD). (**A-C**) Three independent qPCR runs, each performed with three technical replicates for the 10^1 copies/μL and 10^0 copy/μL templates. Dashed lines represent the threshold.
